# Evaluating the utility of mid-infrared spectral subspaces for predicting soil properties

**DOI:** 10.1016/j.chemolab.2016.02.013

**Published:** 2016-04-15

**Authors:** Andrew M. Sila, Keith D. Shepherd, Ganesh P. Pokhariyal

**Affiliations:** a World Agroforestry Centre (ICRAF), P.O. Box 30677-00100 GPO, Nairobi, Kenya; b School of Mathematics, University of Nairobi, P.O Box 30196-00100 GPO, Nairobi, Kenya

**Keywords:** Subspace, Cosine, Hit quality index, Archetypes, Self-organizing maps, Spectroscopy

## Abstract

We propose four methods for finding local subspaces in large spectral libraries. The proposed four methods include (a) cosine angle spectral matching; (b) hit quality index spectral matching; (c) self-organizing maps and (d) archetypal analysis methods. Then evaluate prediction accuracies for global and subspaces calibration models. These methods were tested on a mid-infrared spectral library containing 1907 soil samples collected from 19 different countries under the Africa Soil Information Service project. Calibration models for pH, Mehlich-3 Ca, Mehlich-3 Al, total carbon and clay soil properties were developed for the whole library and for the subspace. Root mean square error of prediction was used to evaluate predictive performance of subspace and global models. The root mean square error of prediction was computed using a one-third-holdout validation set. Effect of pretreating spectra with different methods was tested for 1st and 2nd derivative Savitzky–Golay algorithm, multiplicative scatter correction, standard normal variate and standard normal variate followed by detrending methods. In summary, the results show that global models outperformed the subspace models. We, therefore, conclude that global models are more accurate than the local models except in few cases. For instance, sand and clay root mean square error values from local models from archetypal analysis method were 50% poorer than the global models except for subspace models obtained using multiplicative scatter corrected spectra with which were 12% better. However, the subspace approach provides novel methods for discovering data pattern that may exist in large spectral libraries.

## Highlights

• Global model performed better than local models.
• MSC preprocessed spectra gave poor models, 1st and 2nd derivative spectra gave stable models.
• Mehlich-3 calcium gave the highest coefficient of variation.
• Total carbon global and subspace models using first derivative preprocessed spectra are similar.

## 1 Introduction

Infrared spectroscopy is providing soil scientists with a new tool for assessing soil quality rapidly and cheaply and is opening up new possibilities for monitoring soil quality in landscapes [33], [41] and for digital soil mapping. Other applications of soil spectroscopy have also emerged, for instance, using infrared spectroscopy method as a tool for inferring past climatic changes from lake sentiments determination of organic matter fractions, assessment and monitoring soil quality [9]. Although near-infrared (NIR) and mid-infrared (MIR) spectroscopy are the commonly used techniques for soil measurements Attenuated Total Reflectance (ATR) and Raman spectroscopy approaches have been shown to be useful. Jahn et al. [17] used ATR methods to assay nitrates and ammonium ions from soil. Raman has been used for soil classification studies and recently for assessing the structural role of copper in the soil active glasses [35]. Among the different spectroscopy techniques NIR and MIR are the low-cost and easy to use and have successfully been used to measure carbon (C) content [4]. The main difference between the two ranges is that absorption in mid-infrared spectroscopy corresponds to fundamental bands of molecular vibrations, whereas near-infrared absorptions are due to overtones and combinations of overtones according to several articles cited by Bellon-Maurel and McBratney [4]. Although NIR requires less sample preparation than MIR makes it best suited for in-field analysis but with advancing technology new portable MIR instruments are emerging which can be used in the field giving better specificity and reproducibility of spectra. Because of the dominant intensive vibrations found in MIR spectra, it is generally believed to be more powerful than NIR [20]. Ludwig's view is supported by Pirie et al. [26] who reported a better performance for MIR correlation coefficients validation sets: 0.79 ≤ r ≤ 0.92 against those of NIR 0.53 ≤ r ≤ 0.87 for pH, organic C, clay, sand Mehlich-3 Ca and Mg in Australian top and sub soils.

As spectroscopy instruments continue to improve and scientists' confidence in the usefulness of spectroscopy methods increase as evidenced by scaling up from characterizing few soil samples collected in one site to regionally based land assessments and monitoring studies [37] the need for robust soil spectral prediction models has risen. A result of the increased sampling and subsequent collection of spectra has given rise to the development of large spectral libraries. Generating spectral libraries with wide spectral diversity has been recommended for building calibration models that can reliably be used to predict spectra recorded from new samples [32].

Following an earlier work as discussed in Brown et al. [7] showed that about 5.2 × 10^9^ carefully selected calibration samples will be required to span the global soil compositional space. However, using the large amounts stored in the spectral libraries can decrease the accuracy of the models fitted to predict multiple soil attributes [27]. This is due to a large spectral diversity of samples in terms of geographical origin, environmental conditions, parent material, mineralogy, etc. For instance, Viscarra Rossel et al. [40] stated that NIR spectra and soil properties can vary under different soil mineralogy and their content in soil organic matter. Ramirez-Lopez et al. [27]) observed that modeling soil attributes using large and diverse soil infrared spectral libraries remains a challenging task. To utilize the growing spectral libraries several strategies have been proposed to partition the complexities found in global spectral spaces into local spaces using either geographical or spectral partitioning. For instance, Wetterlind and Stenberg [42] used models calibrated with a national visible-NIR library, and models calibrated only with local samples grouped according to fields sampled. They observed that the local models outperformed the national calibration models. Stevens et al. [34] observed after partitioning soil dataset into different soil texture classes and agro-pedological regions that local NIR models of soil organic carbon perform better than global models. Spectral space partitioning has been done using memory-based learning methods, which search, through a spectral library to find similar spectra. A recent work done by Dahlbacka et al. [10] presented a proof of concept study for using an iterative algorithm to find local quantitative PLS. In their study, they compared different methods for calculating similarity measures for refining the models by removing a specified number of calibration spectra that represent constituent values from the predicted value, then created a new PLS model on the reduced calibration set to make a new prediction. However these approaches are computationally intensive and the criteria for searching through a spectral library and identify points in a local neighborhood have not been satisfactorily developed.

In this study, we proposed and developed simple methods for partitioning global spectral library space into subspaces from which local calibration models will be developed and assessed against global models. We are proposing four different methods for identifying the spectral subspaces:

1. spectral matching using absolute value algorithm to calculate hit quality index value for a spectral library,
2. spectral matching using spectral correlation algorithms to compute pairwise cosine angles,
3. use of self-organizing maps to group spectra into subspaces equal to the number of subspaces obtained using pure mineral matching and
4. archetype analysis of spectra.

### 1.1 Local spectral spaces methods

#### 1.1.1 Cosine spectral similarity angle

Cosine of the angle between two vectors can be used to express similarity between two spectra and has been used extensively in NIR mathematical treatment for expressing sample similarity [6] and can be derived using the Euclidean dot product formula as follows:

(1) $$a.b=\left\|a\right\|\left\|b\right\|\text{cosθ}.$$

With two vectors holding measurements for two samples, A and B, the cosine similarity, cosθ, is represented using a dot product and magnitude as:

(2) $$\text{similarity}=\text{cosθ}=\frac{A.B}{\left\|A\right\|\left\|B\right\|}=\frac{\sum_{i=1}^{n}A_{i}\times B_{i}}{\sqrt{\sum_{i=1}^{n}{\left(A_{i}\right)}^{2}}\times \sqrt{\sum_{i=1}^{n}{\left(B_{i}\right)}^{2}}}.$$

The resulting similarity metric measure ranges from − 1 meaning exactly opposite, to 1 meaning similar vectors with 0 indicating orthogonality (dissimilarity) and values in between indicating intermediate similarity between the vectors. In its application the correlation thresholds obtained using this method are used to determine whether two spectra are a match, and which the correlation is an angle and not a probability. Thus, a threshold of 0.78 in no way means 78% likelihood or 78% confidence.

#### 1.1.2 Hit quality index (HQI)

Spectral library matching is a widely used interpretation aid [47] in spectroscopy applications. The idea behind spectral matching is to mathematically compare unknown (or a new sample) spectrum against a collection of known spectra. The result of this comparison is a number called the “hit quality index”, (HQI) which is a direct measure of how similar two spectra are to each other. To increase the odds of obtaining an accurate search, it is advisable to use the full spectrum. A typical spectrum contains thousands of data points. Different search algorithms are available for comparing two spectra to each other depending on the software being used. In the nomenclature of spectral library searching, the similarity of two spectra can be defined to as a normalized measure of spectral covariance:

(3) $$\mathrm{HQI}=\frac{{\left(\text{known}.\text{unknown}\right)}^{2}}{\left(\text{known}.\text{known}\right)\left(\text{unknown}.\text{unknown}\right)}.$$

Where known denotes the spectrum of a reference material whose identity of either chemical or physical composition is known, unknown denotes the spectrum of the material under investigation or the sample being compared with the known spectra, and the dot symbolizes the dot product of two spectral vectors.

Another simple search algorithm for computing hit quality index is what is referred to as absolute value algorithm. First, a known or reference spectrum is subtracted from the sample spectrum. The result of this calculation is called a residual. The size of residual is directly related to how similar two spectra are to each other. For example, two identical spectra will produce a residual of zero almost a straight line. We have implemented this method in our study due to its simplicity.

#### 1.1.3 Self-organizing maps (SOMs)

Self-organizing map (SOM) belongs to a category of Artificial Neural Networks (ANN) called competitive learning networks. The first author of SOM Teuvo Kohonen [21], simply defines the methods as map reflecting topological ordering. SOM uses a lattice L of n neurons. The arrangement and weights of the neurons are determined by the input set Χ ⊆ ℝ^*d*^ and an updating/training algorithm. The design of the algorithm is such that it positions the neurons within the neuron space in a way to preserve both distribution and topology. During the training process, a weight vector *w*_*i*_ ∈ ℝ^*d*^ is assigned to each neuron *i* ∈ *L*. The weights are also referred to as “prototypes” or “codebook” vectors. Further, the vector *w*_*i*_ represents the position of the *i*^th^ neuron in the feature space. Each datum is mapped onto a neuron associated with the nearest weight vector, e.g. the one with the smallest Euclidean distance from the data pattern, but any other similarity metric can be used. Finding the best-matching unit (BMU) is considered the most computational and important tasks associated with a SOM algorithm. The SOM organizes itself during a competitive and unsupervised learning process. Each pattern is shown to the SOM (randomly or sequentially) and the closest node becomes the “winner”. The learning process yields a map $G_{L}=\left(\Phi_{L\to X},\Phi_{X\to L}\right)$, which, defines two mappings, and functions from an input vector *x* ∈ Χ to a neuron *i* ∈ *L* with a particular weight vector *w*_*i*_ ∈ Χ. The two mappings are defined as follows [1]:

(4) $$G_{L}=\left\{\begin{array}{c} \Phi_{L\to X}:Χ\to L;\;X\in Χ\mapsto d\left(X\right)\in L\\ \Phi_{X\to L}:L\to Χ;\;\in L\mapsto w_{i}\in Χ \end{array}\right).$$

Where *d*(*x*) corresponds to the neuron, which is closest to the *i*^th^ datum. A typical SOM algorithm can be summarized as:

SOM algorithm

This technique has been widely applied in disciplines dealing with high-dimensional in the area of machine vision and image analysis, optical character and script reading, speech analysis and recognition, health, signal processing and radar measurements, industrial and other real-world measurements, process control, mathematical problems and artificial intelligence problems [1]. Most of the past evaluation of SOMs' performance focused on comparisons with other techniques, such as principal component analysis and k-means clustering [23] and while in another work for developing a SOM toolbox [39] involved performance test where computational requirements of the algorithms, i.e., computing time for different training methods, not the quality of the mapping or the reliability of the classes mapped. In this study, the SOM algorithm output will be determined by assessing the type of spectral signatures grouped together into the local subspace.

#### 1.1.4 Archetypes

Archetype analysis has the aim to represent observations in a multivariate data set as convex combinations of extreme points [13]. Consider  *n* × *p* matrix *X* representing a multivariate data set with *n* observations and *p* variables. The goal of archetypal analysis is to find *k* × *p* matrix *Z* that characterizes the archetypal patterns in the data, such that data can be represented as mixtures of those archetypes. Precisely, the archetypal analysis aims at obtaining the two *n* × *k* coefficient matrices *α*  and *β*, which minimize the residual sum of squares:

(5) $$\mathrm{RSS}={\left\|X-\alpha Z^{T}\right\|}_{2}.$$

The elements are required to be greater or equal to zero and their sum must be 1, i.e., $\sum_{j=1}^{k}\alpha_{\mathrm{ij}}=1$ with *α*_*ij*_ ≥ 0 and *i* = 1 . *n* . ‖.‖_2_ denotes an appropriate matrix norm.

The archetypes are convex combinations of the data points:

(6) $$Z=X^{T}\beta .$$

Where, *β* is the second set of coefficients of the data set, *n* × *k* is a matrix whose elements are required to be greater or equal to zero and their sum must be 1, i.e., $\sum_{i=1}^{n}\beta_{\mathrm{ij}}=1$ with *β*_*ji*_ ≥ 0 and *j* = 1 … *k*.

These two fundamentals also define the basic principles of the estimation algorithm: it alternates between finding the best *α* for given archetypes *Z* and finding the best archetypes *Z* for given *α*; at each step several convex least squares problems are solved, the overall RSS is reduced successively [14].

### 1.2 Spectral pretreatment methods

#### 1.2.1 Multiplicative scatter correction

Multiplicative scatter correction (MSC) was proposed by Isaksson and Naes [16] to correct for light scattering or change in path length for each sample estimated relative to that of an ideal sample. In principle this estimation should be done on a part of the spectrum that does not contain chemical information, i.e. influenced only by the light scattering. However, the areas in the spectrum that hold no chemical information often contain the spectral background where the signal to noise (SNR) may be poor. In practice, the whole spectrum is sometimes used. This can be done provided that the chemical differences between the samples appear to have the same scatter level as the ideal. As an estimate of the ideal sample, we can use for instance the average of the calibration set. MSC performs best if an offset correction is carried first. For each sample:

(7) $$X_{i}=a+b{\overset{®}{X}}_{j}+e.$$

Where *X*_*i*_ is the NIR spectrum of the sample, and ${\overset{®}{X}}_{j}\;$ symbolizes the spectrum of the ideal sample (the mean spectrum of the calibration set). For each sample, *a* and *b* are estimated by ordinary least-squares regression of spectrum *X*_*i*_  versus ${\overset{®}{X}}_{j}\;$ spectrum over the available wavelengths. Each value *X*_*ij*_  of the corrected spectrum *X*_*i*_ (MSC) is calculated:

(8) $$X_{\mathrm{ij}}\left(\mathrm{MSC}\right)=\frac{X_{\mathrm{ij}}-a}{b};j=1,2,\ldots ,p.$$

#### 1.2.2 Standard normal variate

Standard normal variate (SNV) has been proposed for removing the multiplicative interference of scatter and particle size [3]. The SNV transformation centers each spectrum and then scales it by its own standard deviation:

(9) $$X_{\mathrm{ij}}\left(\mathrm{SNV}\right)=\frac{X_{\mathrm{ij}}-{\overset{®}{X}}_{i}}{\mathrm{SD}};j=1,2,\ldots ,p.$$

Where *X*_*ij*_  is the absorbance value of spectrum *i*  measured at wavelength *j*, ${\overset{®}{X}}_{i}$ is the absorbance mean of the uncorrected in the spectrum and SD is the standard deviation of the *p* absorbance values,

(10) $$\sqrt{\frac{\sum_{j=1}^{p}{\left(X_{\mathrm{ij}}-{\overset{®}{x}}_{i}\right)}^{2}}{p-1}}.$$

Spectra treated in this manner have always zero mean and variance and variance equal to one, and are thus independent of original absorbance values.

#### 1.2.3 De-trending

De-trending of spectra accounts for the variation in baseline shift and curvilinearity of powdered or densely packed samples by using a second-degree polynomial to correct the data [3]. De-trending operates on individual spectra. The global absorbance of NIR spectra is generally increasing linearly with respect to the wavelength, but it increases curvilinearity for the spectra of densely packed samples. A second-degree polynomial can be used to standardize the variation in curvilinearity:

(11) $$X_{i}=a\lambda^{*2}+b\lambda^{*}+c+e_{i}.$$

Where *X*_*i*_  symbolizes the individual IR spectrum and *λ*^∗^  the wavelength. For each sample, *a* , *b*  and *c* are estimated by ordinary least squares regression of spectrum *X*_*i*_(DTR)  is calculated by:

(12) $$X_{i}\left(\mathrm{DTR}\right)=X_{i}-a\lambda^{*2}-b\lambda^{*}-c=e_{i}.$$

Normally de-trending is used after SNV transformation. It has been demonstrated that MSC and SNV transformed spectra are closely related and that the difference in prediction ability between these methods seems to be fairly small [11], [15].

#### 1.2.4 Saviztky–Golay derivatives

Noise within spectral data can be removed by Savitzky–Golay smoothing [31]. In this method, a polynomial least-squares fit is performed on a spectral window around spectral point *j* of *i*^th^ sample. The corrected spectral point (*x*_*ij*_^new^)  is estimated using this calculated polynomial model. Subsequently, the window is shifted to a spectral point (*j* + 1), and the procedure is repeated until the entire spectral range is smoothed. Savitzky–Golay smoothing is also used in combination with 1st and 2nd derivatives from the spectral data [36].

## 2 Materials and methods

### 2.1 Spectral library

To test our approach for determining spectral subspaces, we used MIR spectral libraries from Africa Soil Information Service (AfSIS) project covering sub-Saharan Africa region.

### 2.2 Field sampling

**Fig. 1:**
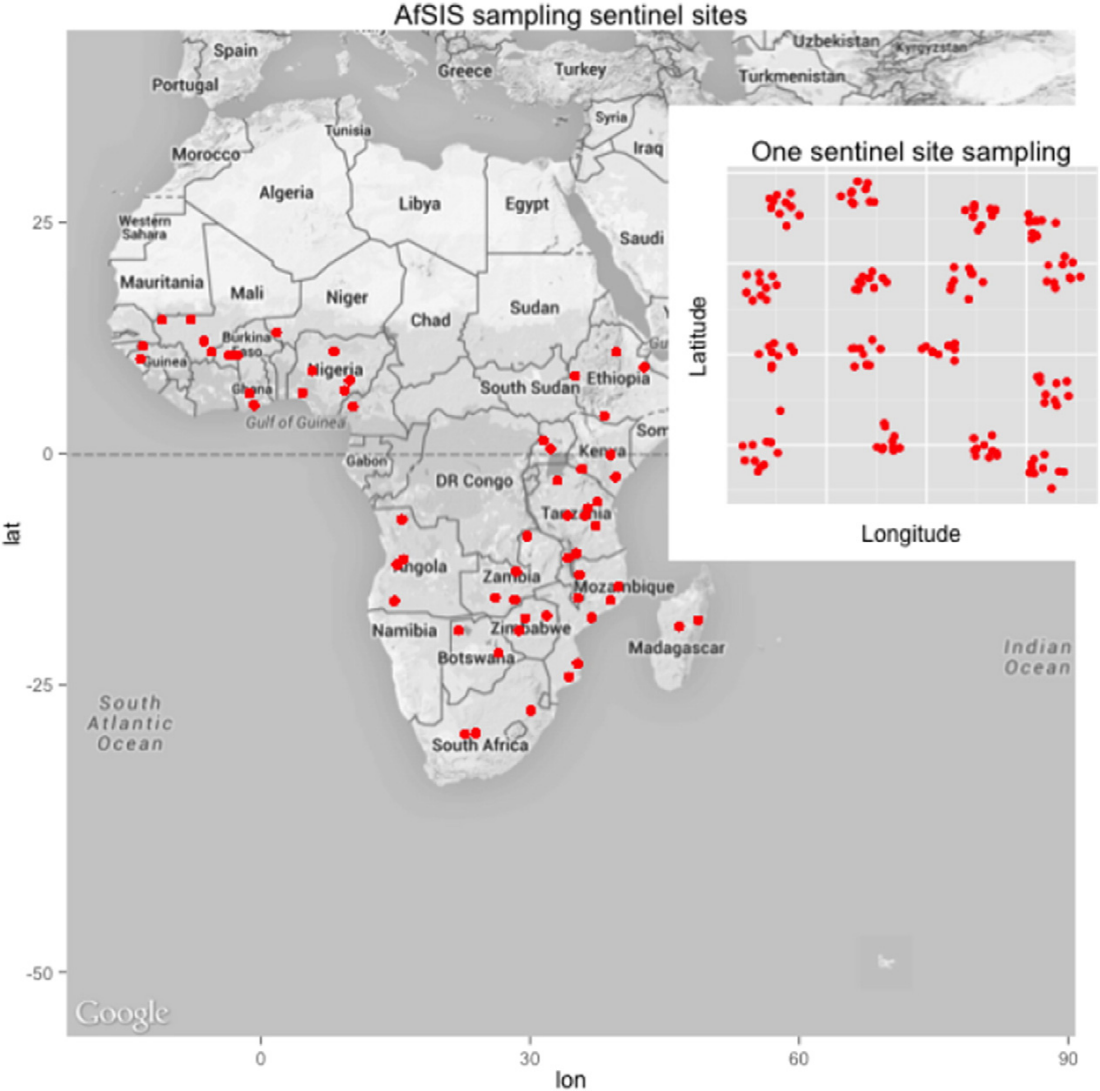
Map of Africa showing the 60 AfSIS sampling sentinel sites in sub-Saharan Africa. Inset plot shows the distribution of sampling points with one sentinel site.

**Fig. 2:**
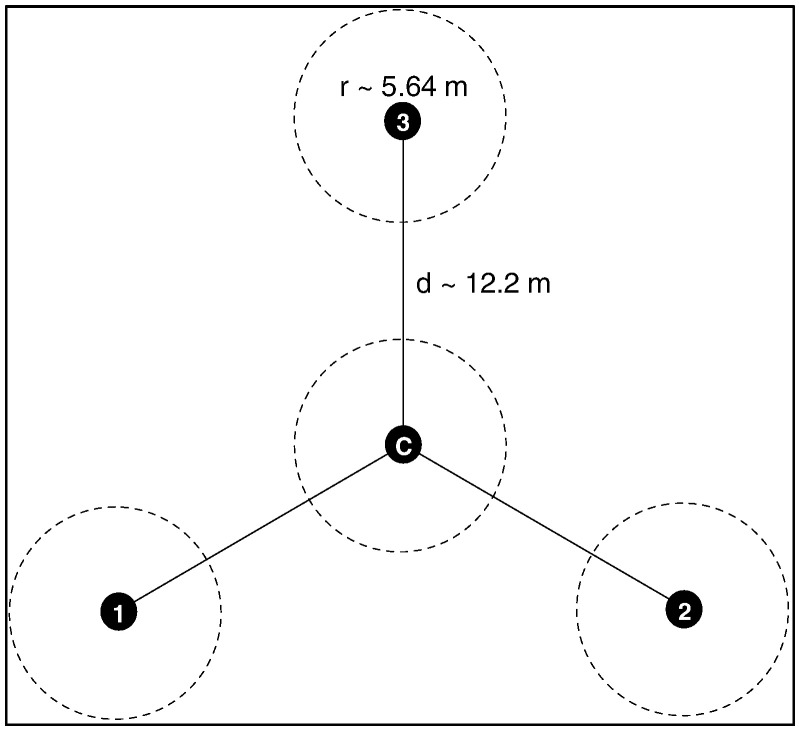
ICRAF LDSF sampling layout at plot and subplot levels. The black dots indicated soil-sampling locations; larger (dashed) circles represented 0.01 ha sub-plots in which soil surface and vegetation observations were carried out. *r* was the subplot radius, and *d* was the center-point distance.

Sampling for AfSIS library was carefully executed to obtain representative soil samples covering approximately 18.1 million km^2^ of the non-desert, including Madagascar [46]. To achieve this 60, 10 × 10 km sized “Sentinel Sites”, stratified by the major Koppen–Geiger climate zones of Africa [25], excluding some of the African countries which were no-go zones due to security reasons were used. Each sentinel site was subdivided into 16 sampling units (clusters), each cluster was further split into 10 smaller sampling units (plots). The sampling plot was designed to sample approximately 1000 m^2^ (0.1 ha or 30 ∗ 30 m) area Fig. 1. Longitude and latitude coordinates were generated for each plot and saved into a Geographical Positioning System (GPS) unit. Field crewmembers easily navigated the geo-referenced plots with the help of the GPS unit but when a point led to a difficult point to sample an alternative plot was established nearby and the new coordinates are saved into the GPS unit. Within a plot, four subplots were identified. To determine the subplot layouts [43], one field crewmember stood at the center marked as subplot 1 as shown in Fig. 2*.*

In the general direction of downslope, subplot 2 was marked at 12.2 m. To mark the upslope sub-plots 3 and 4 (wings of the Y-frame in Fig. 2), the field crewmember standing at subplot 1 broadcast his outstretched hands backwards facing the downslope subplot 2 with the measuring tape at the end of the hand, pulled back the tape to the center of his chest and marked the position of the lefthand side subplot 4 at 12.2 m. The stretching approximated 120° the angle between the subplots. Similarly, the crewmember pulled back the tape to the center of his chest and marked the position of the right handside subplot 3 at the same length of 12.2 m. Four pegs each about 1 m lengths were prepared and labeled 1, 2, 3, and 4. These pegs were used for marking the center points of the subplots. Using a soil auger samples were collected at 0–20 cm and 20–50 cm from the four subplots then composited to give a representative plot sample for each depth.

### 2.3 Laboratory analysis

First all soil samples were air-dried and then large clods were crushed to pass through a 2 mm sieve. All samples received in the laboratory were analyzed for MIR spectra and 10% of the samples were subjected to reference analysis using wet chemistry for a wide range of soil properties but for this study we focus on pH, Mehlich-3 Aluminum (m3.Al), Mehlich-3 Calcium (m3.Ca), total carbon, clay and sand.

#### 2.3.1 Soil sample analysis using wet chemistry methods

The selected samples for reference analysis were thoroughly mixed before scooping. This was to ensure that a homogenous subsample was selected and a similar one was left in the bag, which was to be used for MIR analysis. Soil property analysis by wet chemistry methods was done according to the methods described by Awiti et al. and Brown et al. [2], [7].

#### 2.3.2 MIR spectral measurements and pretreatments

The soil samples were air-dried and then finely ground to powder (approximately < 100 μm) using a sample mill. The pure minerals were also finely ground. The fine samples were then loaded into aluminum microtiter plates (A752-96, Bruker Optics, Karlsruhe) using a micro spatula to fill the 6-mm diameter wells and leveled. Samples were loaded into four replicate wells, each sample was scanned 32 times in MIR reflectance mode using a Fourier-transform MIR spectrometer (FT-IR; Tensor 27, Bruker Optics, Karlsruhe, Germany) with a high throughput screening extension arm using a liquid Nitrogen cooled HgCdTe detector. A single spectrum obtained for each sample was later transferred to a desktop computer where it was converted and combined into a single flat data table.

### 2.4 Pure minerals spectra

A total of 11 different pure mineral types were scanned on the FT-IR Tensor 27 spectrometer. The eleven mineral samples include (i) Biotite; (ii) Chlo; (iii) Halloysite; (iv) Illite; (v) kaolinite; (vi) Montmorillonite; (vii) Muscovite; (viii) Nontronite; (ix) Palygorskite; (x) quartz; and (xi) white-sand. In their natural occurrence, these minerals are some of the most dominant within soils. The spectra obtained were then used as the reference point for subspaces and matched against soil spectra from the library collection. Out of the eleven, Halloysite, quartz, Illite and Montmorillonite were matched with soil spectra in the spectral library.

Halloysite and quartz were obtained from James Hutton institute mineral collection while Illite and Montmorillonite were ordered from the Clay Mineralogical Society.

### 2.5 Spectral subspaces and calibration models

First, the two spectral libraries were split into a training set (70%) and a testing set (30%) of the total number in each library using conditioned Latin hypercube sampling algorithm as implemented in ‘clhs’ R package [28]. This selection was carefully done to ensure that samples from the same sampling point are kept together i.e., topsoil and subsoil from the same sampling point were either in the training or testing set. The combined spectra were preprocessed using (1) Savitzky–Golay 1st and 2nd derivatives with a smoothing interval of 21 points [37]; (2) standard normal variate (SNV); (3) SNV + Detrending; and (4) multiplicative scatter correction (MSC). Infrared data often contain systematic variation like an additive or multiplicative offset, which may be caused by scatter effects due to differences in particle sizes, chemical interferences, or instrument drift. The preprocessing eliminates or reduces the impact of the non-relevant spectral information and often leads to simpler and more robust calibration models. These variations may complicate data analysis and interpretation.

Each set of the preprocessed spectra was used to generate spectral subspaces using the four methods aforementioned.

#### 2.5.1 Spectral cosine angle correlation spectral subspaces (CACSS)

Using the preprocessed spectra, each sample spectrum was projected to one pure mineral spectrum at a time to determine the cosine angle between the two spectra vectors. The pure mineral giving the smallest angle was used to label the sample spectra to belong to the same subspace with that pure mineral. From trigonometry two similar vectors will have an angle of zero degrees between them and taking their cosine gives one. Similarly, the angle between two vectors will widen depending on how the two vary from one another.

#### 2.5.2 Hit quality index spectral subspaces (HQISS)

Here is how we implemented a simple search algorithm, the absolute value algorithm or the hit quality index spectral subspaces (HQISS), to obtain sample spectral library subspaces matching with the spectra for the 11 pure minerals:

1. Pick one actual soil spectrum from the spectral library then subtract from each of the 11 pure minerals' spectra.
2. The result of the subtraction gives a residual, where the size of the residual is directly related to how similar two spectra are to each other. Identical spectra will have a residual equal to zero (a straight line). Dissimilar spectra give residuals less than or greater than zero.
3. Calculate the size of the residual by taking the absolute value of each data point in the residual, take their sum and then divide by the number of data points to get the HQI.
4. Rank the 11 HQIs' to get the lowest value for which corresponds to the pure spectra most similar to the sample.
5. Repeat 1 to 4 for each sample in the library.
6. Identify the subspaces where each spectrum in the library is mapped.

#### 2.5.3 Self-organizing maps spectral subspaces

Excluding the pure minerals' spectra, the samples' spectra table was subjected to a self-organizing map (SOM) analysis to determine the subspaces formed by spectral features according to their similarities. The number of maps fit was decided based on the results obtained from spectral matching using CACSS and HQISS methods.

#### 2.5.4 Archetype spectral subspaces

The hardest part in archetypal analysis is picking on the optimal or best number of archetypes. If prior information is available to the analyst to know the relevant archetypes contained in a particular dataset the known number is used otherwise elbow criterion of a residual function [14] which is the value corresponding to a minimum residual sum of squares (RSS) is used. We fitted four archetypes based on the results obtained from HQISS and CACSS, which also gave a reasonable value corresponding to the minimum RSS.

### 2.6 Model development

Random forest regression was used to calibrate spectra with pH, m3.Al, m3.Ca, total carbon, clay and sand. Global and local models were developed using spectra processed with the five methods explained above. The choice of RF method among the commonly used machine learning methods like partial least regression (PLSR) and principal component regression methods was based on its excellent ability to pick non-linearity relationship between predictors and response variables. Also, it has been reported to be simple in theory, fast speed when handling large data, fine-tuning mechanism to control over-fitting and that it contains an automatic compensation mechanism on biased sample numbers of groups during the training process. Each sample in the testing set was predicted using a calibration model from a corresponding training set i.e. spectra from the same subspace and preprocessed using the same method. There are a number of methods used to assess model performance using test data. The commonly used methods include bias, root mean square error (RMSE) and the ratio of performance (RPD). Because the three statistics will often give similar information leading to the same conclusion, in this study we used RMSE values. Eq. (10) gives the formulae for calculating RMSE where *y* denotes the measured value and $\hat{y}$ the predicted value, *n* is the number of samples and *SD* is the standard deviation of the laboratory-measured value for the soil property being predicted.

(10) $$\text{RMSE}=\sqrt{\frac{1}{n}\sum_{i=1}^{n}{\left(y_{i}-{\hat{y}}_{i}\right)}^{2}}.$$

## 3 Results and discussion

### 3.1 Soil characteristics of global and local models

Descriptive statistics of the soil properties conventionally analyzed in the laboratory for different spectral subspaces are shown in Table 1, Table 2, Table 3, Table 4 for both calibration and validation data. Calibration soil samples are very diverse with soils ranging from very acidic to alkaline soils with pH values ranging from 3.61 to 9.86 but soil samples in each spectral subspace obtained gave narrower ranges. Soil samples in subspace 3 obtained with spectral archetype analysis had a pH range of 7.71–8.86 but a broad range of total carbon of 1.12–11.29%. In terms of soil texture, the samples vary from very sandy to very clay soils with equally high mean values for Al (821 ppm) and Ca (1842 ppm). Variation of the soil properties in different spectral subspaces is varying depending on how well the subspaces classified similar soil samples. For instance, CACSS and archetypes based subspaces are similar in terms of variations in clay content with most of their subspaces giving the highest standard deviation > 20%. Coefficient of variation (%CV) values show m3.Ca had the largest variability of > 100 for archetype4 subspace in the calibration data with a similar %CV obtained from archetype1 of validation m3.Ca. pH %CV for both calibration and validation subspaces was the lowest (2.4–12.5).

**Table 1:** Summary of soil properties for both calibration and independent validation set for SCC spectral subspace.

Subspace	Variable	Units	n	Min	Max	Mean	s.d	%CV	n	Min	Max	Mean	s.d	%CV
Halloysite														
	Clay	%	65	3.67	97.1	44.7	23.1	51.7	22	8.75	95.83	67.96	28.2	41.5
	m3.Al	ppm	65	16.3	2538	932.6	550.8	59.1	22	176	1740	1057.95	373.9	35.3
	m3.Ca	ppm	65	42.61	16,823.1	1842.2	3050.7	165.6	22	69.9	2310	564.92	666.8	118.0
	pH	–	65	4.19	8.78	6.3	1.4	22.2	22	4.3	6.85	5.15	0.8	15.5
	Sand	%	65	0.79	93.8	37.8	25.5	67.5	22	0.12	87.51	17.18	29.1	169.4
	Totalcarbon	%	65	0.14	7.53	1.5	1.6	106.7	22	0.14	5.22	2.14	1.6	74.8
Illite														
	Clay	%	418	1.34	99.27	44.2	23.8	53.8	173	3.3	100	38.99	23.5	60.3
	m3.Al	ppm	418	1.67	2664	852.2	472	55.4	173	60.9	1700	713.44	396.1	55.5
	m3.Ca	ppm	418	58.2	35,200	2711.4	5256.5	193.9	173	9	11,000	836.26	1373.7	164.3
	pH	–	418	3.61	9.21	6.4	1.1	17.2	173	4.18	9.86	5.89	1	17.0
	Sand	%	418	0.12	98.66	38.3	25.6	66.8	173	4	93.89	45.42	27.4	60.3
	Totalcarbon	%	418	0.14	11.29	1.4	1.5	107.1	173	0.08	6.32	0.75	0.7	93.3
Montmorillonite														
	Clay	%	555	0.32	97.3	45	23.9	53.1	176	4.53	100	42.4	27.3	64.4
	m3.Al	ppm	555	19.36	3041	904.7	503	55.6	176	14.3	2240	859.69	518.4	60.3
	m3.Ca	ppm	555	29.26	17,600	2128.6	3148	147.9	176	0	9960	863.6	1409.6	163.2
	pH	–	555	4	9.24	6.4	1.1	17.2	176	4.01	8.87	5.86	0.9	15.4
	Sand	%	555	0.68	99.68	37.9	26.1	68.9	176	0.43	100	42.08	29.3	69.6
	Totalcarbon	%	555	0.11	10.66	1.4	1.4	100.0	176	0.12	9.4	1.21	1.4	115.7
Quartz														
	Clay	%	291	2.21	93.6	43.3	22.3	51.5	204	6.8	90.8	35.06	22.3	63.6
	m3.Al	ppm	291	15.4	1960	771.6	366.1	47.4	204	87.3	1850	601.94	362.2	60.2
	m3.Ca	ppm	291	66.3	24,700	1849.9	3181.7	172.0	204	40.1	9690	1091.41	1482.8	135.9
	pH	–	291	4.18	9.27	6.1	0.9	14.8	204	4.57	9.72	6.21	0.9	14.5
	Sand	%	291	1.38	96.79	35.1	22.7	64.7	204	1.38	90.06	48.39	25.2	52.1
	Totalcarbon	%	291	0.11	7.09	1.3	1.2	92.3	204	0.1	6.67	0.81	1	123.5

**Table 2:** Summary of soil properties for both calibration and independent validation set for hit quality index spectral subspaces.

Subspace	Variable	Units	n	Min	Max	Mean	s.d	%CV	n	Min	Max	Mean	s.d	%CV
Halloysite														
	Clay	%	13	35.26	92.72	78.3	16.6	21.2						
	m3.Al	ppm	13	345.8	2242	1309	520.4	39.8						
	m3.Ca	ppm	13	133.3	1011	469.7	249.2	53.1						
	pH	–	13	5.21	6.38	5.8	0.3	5.2						
	Sand	%	13	2.71	21.9	10.1	6	59.4						
	Totalcarbon	%	13	0.11	1.69	0.6	0.4	66.7						
Illite														
	Clay	%	1098	1.1	99.27	45	22.5	50.0	418	8.53	100	46.4	22.7	48.9
	m3.Al	ppm	1098	1.67	2722	898.1	474.5	52.8	418	14.3	2240	867.5	428.9	49.4
	m3.Ca	ppm	1098	29.26	35,200	2170.8	3995.8	184.1	418	0	11,000	943.4	1420.1	150.5
	pH	–	1098	3.61	9.27	6.3	1.1	17.5	418	4.01	9.43	5.9	1	16.9
	Sand	%	1098	0.12	98.9	36.7	24.5	66.8	418	0.12	100	35.2	22.9	65.1
	Totalcarbon	%	1098	0.13	11.29	1.5	1.5	100.0	418	0.08	9.4	1.2	1.2	100.0
Montmorillonite														
	Clay	%	90	10.96	97.3	65.3	18.9	28.9	24	25.71	100	72.8	15.9	21.8
	m3.Al	ppm	90	424	3041	925.9	388.2	41.9	24	430	1530	760.8	281.2	37.0
	m3.Ca	ppm	90	104	14,858.3	5961.7	4005.6	67.2	24	1130	7570	3704.6	1634.5	44.1
	pH	–	90	4.39	8.98	7.3	1.1	15.1	24	4.98	9.72	7.1	1.1	15.5
	Sand	%	90	1.65	85.26	18.1	16.6	91.7	24	1.38	67.55	14.5	14.2	97.9
	Totalcarbon	%	90	0.25	2.68	1.1	0.5	45.5	24	0.24	3.22	1	0.6	60.0
Quartz														
	Clay	%	128	0.32	55.71	20.9	11.6	55.5	133	3.3	25.5	12.8	5.3	41.4
	m3.Al	ppm	128	106	1360	445.7	196	44.0	133	60.9	593	300.4	99.7	33.2
	m3.Ca	ppm	128	58.2	1725	364	287.8	79.1	133	31.8	1700	364.6	256.8	70.4
	pH	–	128	4.32	8.05	5.9	0.7	11.9	133	4.72	9.86	6	0.7	11.7
	Sand	%	128	21.27	99.68	59.9	19.5	32.6	133	42.54	93.89	78.3	9.6	12.3
	Totalcarbon	%	128	0.11	1.02	0.4	0.2	50.0	133	0.09	0.95	0.3	0.1	33.3

**Table 3:** Summary of soil properties for both calibration and independent validation set for spectral archetypes subspaces.

			Calibration						Validation					
Subspace	Variable	Units	n	Min	Max	Mean	s.d	%CV	n	Min	Max	Mean	s.d	%CV
Archetype1														
	Clay	%	292	2.22	97.3	52.7	21.5	40.8	86	2.22	88.03	42.2	23.9	56.6
	m3.Al	ppm	292	34	1434	649.7	267.1	41.1	86	47.39	1386	700.4	277.2	39.6
	m3.Ca	ppm	292	514	20,593.1	5533.2	4396.3	79.5	86	629.8	20,593.1	5201.7	5234.7	100.6
	pH	–	292	3.61	9.27	7.6	0.9	11.8	86	5.69	8.59	7.4	0.8	10.8
	Sand	%	292	1.38	96.06	30	22.5	75.0	86	2.56	96.06	40.4	29.4	72.8
	Totalcarbon	%	292	0.19	8.07	1.3	1.1	84.6	86	0.25	8.07	1.5	1.4	93.3
Archetype2														
	Clay	%	393	0.32	74.35	23.6	12.3	52.1	171	0.32	50.43	20.1	11.5	57.2
	m3.Al	ppm	393	106	1660	539.2	225.6	41.8	171	138	1037	520.8	185.7	35.7
	m3.Ca	ppm	393	29.26	3696	533.1	465.3	87.3	171	130.6	3696	634.3	456.7	72.0
	pH	–	393	4.32	8.78	6.1	0.7	11.5	171	4.32	8.16	6.3	0.6	9.5
	Sand	%	393	7.87	99.68	58.6	19.4	33.1	171	15.86	99.68	64.5	18.9	29.3
	Totalcarbon	%	393	0.11	2.92	0.6	0.4	66.7	171	0.11	2.42	0.6	0.3	50.0
Archetype3														
	Clay	%	29	23.76	80.86	51.4	18.2	35.4	20	32.98	80.86	57.5	16.5	28.7
	m3.Al	ppm	29	1.67	419	149.4	136.5	91.4	20	1.67	322.4	165	121.7	73.8
	m3.Ca	ppm	29	6540	35,200	17,824.7	8549.2	48.0	20	8377	34,460	17,299.3	7769.9	44.9
	pH	–	29	7.71	8.86	8.3	0.3	3.6	20	7.81	8.52	8.2	0.2	2.4
	Sand	%	29	2.25	59.16	20.3	14.9	73.4	20	2.25	40.41	14.3	10.9	76.2
	Total carbon	%	29	1.12	11.29	5.8	2.5	43.1	20	3.84	11.29	6.5	2.3	35.4
Archetype4														
	Clay	%	615	1.55	99.27	53.9	21.7	40.3	299	1.55	93.15	49	23.3	47.6
	m3.Al	ppm	615	345.8	3041	1201.8	433.2	36.0	299	485.1	3041	1286.2	500.9	38.9
	m3.Ca	ppm	615	63	8550	987.7	997.9	101.0	299	109	6085.98	1061.3	1018	95.9
	pH	–	615	3.94	8.29	5.7	0.7	12.3	299	4.55	8.07	5.9	0.6	10.2
	Sand	%	615	0.12	97.83	27.7	21.5	77.6	299	1.31	97.83	34.1	25.5	74.8
	Totalcarbon	%	615	0.11	7.4	1.7	1.4	82.4	299	0.24	7.09	1.4	1.3	92.9

**Table 4:** Summary of soil properties for both calibration and independent validation set for self-organizing maps spectral subspaces.

			Calibration						Validation					
Subspace	Variable	Units	n	Min	Max	Mean	s.d	%CV	n	Min	Max	Mean	s.d	%CV
SOM1														
	Clay	%	202	0.32	55.71	19.7	11.2	56.9	172	3.3	69.47	15.5	10.5	67.7
	m3.Al	ppm	202	106	1660	470.6	219.1	46.6	172	60.9	1490	332.1	167.5	50.4
	m3.Ca	ppm	202	29.26	3696	402.4	387.1	96.2	172	0	1700	392.6	276	70.3
	pH	–	202	4.32	8.78	5.9	0.7	11.9	172	4.72	9.86	6.1	0.8	13.1
	Sand	%	202	15.58	99.68	62.7	19.7	31.4	172	8.93	93.89	74.5	14.8	19.9
	Totalcarbon	%	202	0.11	1.58	0.5	0.3	60.0	172	0.09	1.64	0.3	0.2	66.7
SOM2														
	Clay	%	458	1.55	87.61	36.8	17.8	48.4	218	12.13	100	36.9	16.5	44.7
	m3.Al	ppm	458	224	1903	827.8	329.6	39.8	218	271	2120	796.2	378.9	47.6
	m3.Ca	ppm	458	63	5862	763.5	672.1	88.0	218	9	3220	489.2	529.5	108.2
	pH	–	458	3.94	8.76	6	0.9	15.0	218	4.01	9.43	5.7	0.9	15.8
	Sand	%	458	1.59	97.83	46.4	22.1	47.6	218	0.82	78.57	43.2	18.3	42.4
	Totalcarbon	%	458	0.14	6.66	0.9	0.7	77.8	218	0.08	3.16	0.7	0.5	71.4
SOM3														
	Clay	%	439	3.83	99.27	55.9	22.4	40.1	134	9.68	100	70.3	16.4	23.3
	m3.Al	ppm	439	345.8	3041	1224	487.2	39.8	134	430	2240	1064.6	346.4	32.5
	m3.Ca	ppm	439	83.9	8550	1330.4	1208.9	90.9	134	20.8	5290	1368.4	1325.5	96.9
	pH	–	439	4	8.79	6	0.8	13.3	134	4.19	9.72	5.9	0.9	15.3
	Sand	%	439	0.12	93.63	24.4	20.1	82.4	134	0.12	67.55	13.8	12.6	91.3
	Totalcarbon	%	439	0.11	7.4	1.9	1.5	78.9	134	0.21	9.4	1.6	1.3	81.3
SOM4														
	Clay	%	230	4.23	97.3	59.2	19	32.1	51	24.77	100	53.9	17	31.5
	m3.Al	ppm	230	1.67	1640	573.5	321.7	56.1	51	14.3	1940	930.3	550.4	59.2
	m3.Ca	ppm	230	593	35,200	8512.1	6174.1	72.5	51	73.4	11,000	3415.9	2821.3	82.6
	pH	–	230	3.61	9.27	7.9	0.9	11.4	51	4.08	8.89	7	1.3	18.6
	Sand	%	230	1.38	93.29	22.2	17.2	77.5	51	0.97	100	25.9	18.4	71.0
	Totalcarbon	%	230	0.19	11.29	2.1	2	95.2	51	0.49	6.98	2.4	1.9	79.2

Mean distributions of soil properties across the other subspaces were different. In Table 2, we see HQISS put samples with lowest mean carbon (0.4) and highest sand content (78.3) to subspace related with quartz. Samples associated with Illite and Montmorillonite subspaces gave the highest carbon (1.5) and clay (64) respectively. There were only 13 samples associated with Halloysite pure mineral in the calibration of HQISS and none in the validation set. Illite subspace was the most dominant while Halloysite is the least dominant with 13 samples, which were all from the calibration set, and none from the validation. Similar to the archetypal subspaces, m3.Ca within HQISS subspaces gave the highest %CV values > 180.

In Table 3 archetype subspaces seem to have been created based on carbon and soil texture variations. Samples put in archetype3 have the highest carbon (5.8) and the lowest sand (20.3) while archetype2 has the lowest carbon (0.6) but highest sand (58.6). Although archetypes 1 and 4 are rich in clay > 50, they contain varying levels of pH (7.6, 5.7) and Al (649.7, 1201.8) respectively. It is likely that the high Al content in archetype4 contributes to the slight acidity of the samples in this subspace.

Similarly, SOM subspaces appear to have been created on the basis of soil texture and carbon variations with SOM4 subspace giving the highest carbon (2.1) and highest clay content (59.2) while SOM1 has the lowest carbon (0.7) and clay content (19.2). The most dominant spectral subspaces SOM2 and SOM3 consist of samples with highest Al (827, 1224) and equal low pH (6). Due to the high Al concentration and the low (pH ≤ 5.5), the soil samples falling into these SOM subspaces are likely to be acidic. Reyes-Díaz et al. [29] stated that toxic Al^+ 3^ results in a reduction of crop root growth and eventually overall plant toxicity leading to reduced crop yields. The overall variability for SOM subspaces was lowest among the four subspaces considered for m3.Ca %CV values of 82.6–108.2.

As expected, we found that the independent validation set had a similar distribution to the calibration set but with narrower ranges for the six-soil properties. This is a good indicator that the selected validation points fall within the boundary of the calibration space hence increasing the chance of being reliably predicted because they share similar features.

### 3.2 Distribution of MIR spectra within local spaces

**Fig. 3:**
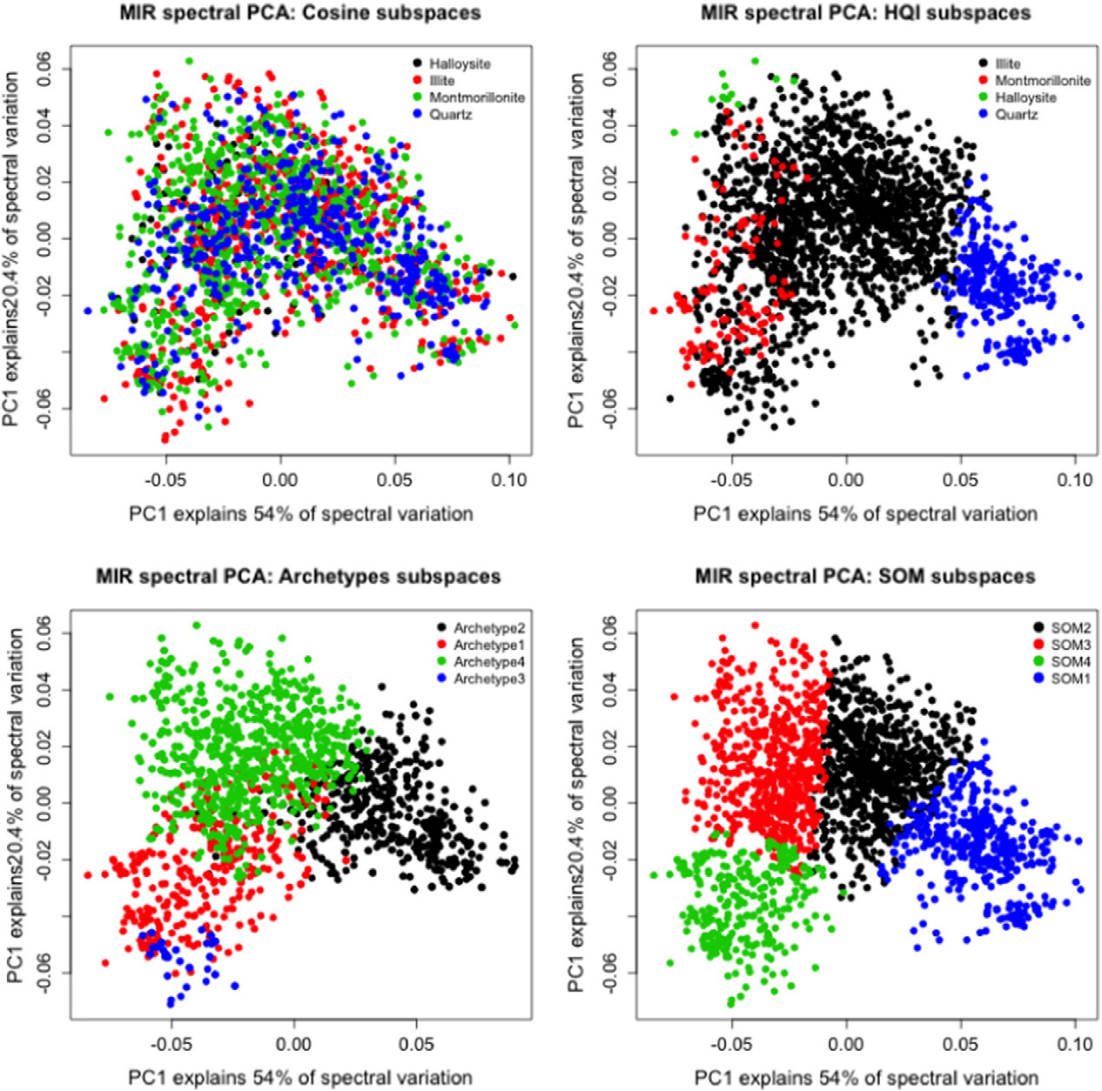
1st derivative preprocessed MIR spectra PCA scores' sample points labeled in each sample space.

Distribution of the samples within their local spaces is shown in Fig. 3 using score plot for the first two principle components (PCs) for all the 1906 samples used in this study. The first two PCs explain up to 74.4% of the original mid-infrared spectral variation, which comprises both physical and chemical soil information. Using different colors and labeling sample points according to their local subspaces, we showed how well some of the subspace methods discovered hidden structure in the global spectral library. For instance, the SOMSS gave well-separated clusters, labeled as SOM1, SOM2, SOM3 and SOM4. When the points were projected into a PC score plot and read side by side with the subspaces from HQISS it was easy to relate SOM1 samples with soil samples identified as close to the sample with pure quartz. Samples associated with SOM2 can be said to belong to sample class associated with pure Montmorillonite mineral. SOM3 gave mixed samples associated with Halloysite, Montmorillonite and Illite pure minerals as identified in the HQISS. SOM4 was also a mixed bag when related to samples identified in both the ArchetypeSS and HQISS. In the ArchetypeSS it is seen to be dominated by archetype1 interspersed with the few samples assigned to archetype-3 and a mixture of samples associated with Montmorillonite and Illite. Using Tukey's test we found that mean total carbon between subspaces obtained using SOMSS and ArchetypeSS differed significantly in each subspace.

**Fig. 4:**
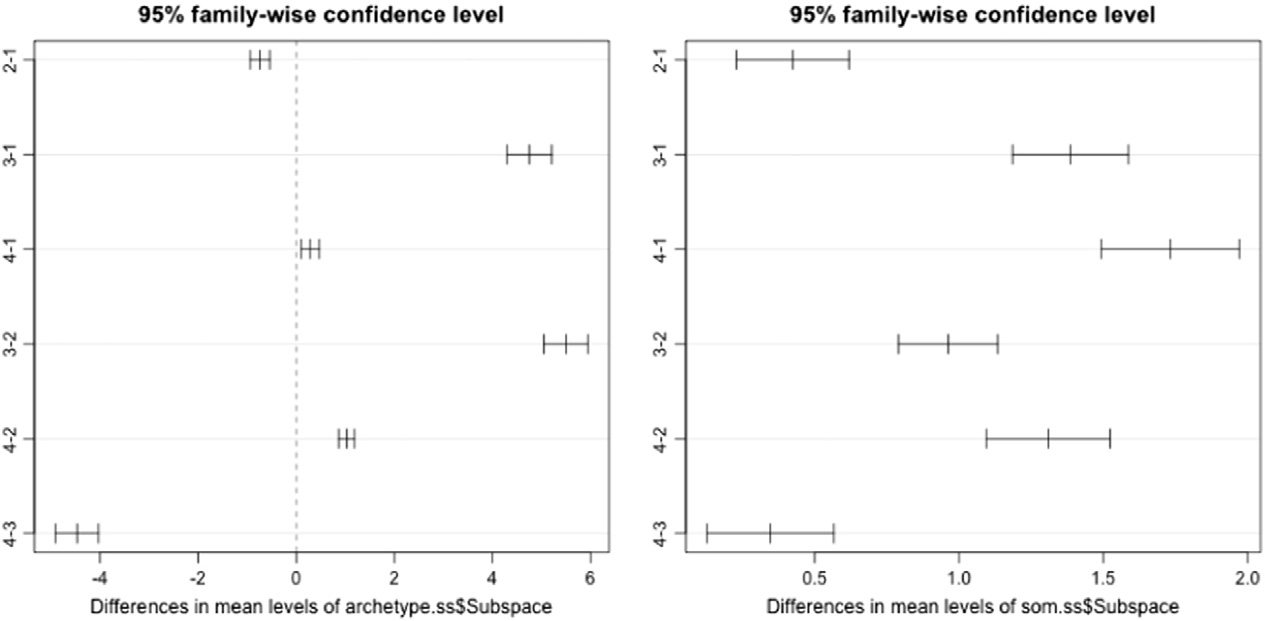
Archetype-SS (left plot) and SOM-SS (right plot) confidence interval plot showing mean soil total carbon (%) differences among the spectral subspaces. All the four subspaces in each type are significantly different.

We used HQISS to understand the common spectral features within a subspace. We averaged all spectra in each subspace and obtained a representative spectrum with different shapes and intensities Fig. 4. Some of the clay minerals found in soil include kaolinite, Halloysite, quartz, carbonate, gibbsite, Illite, and smectite in widely varying proportions [24]. Illite minerals are characterized by a broad and poorly defined hydroxyl stretching band near 3620 and 3630 cm^− 1^ [24]. Illite rich soils are also referred to as desert loam soils and from spectral subspaces obtained they are dominant with about 80% of the samples grouped to be similar to Illite.

Montmorillonite is a subclass of the smectite clay mineral with a prominent absorption band centered at ~ 1639 cm^− 1^ according to Yitagesu et al. [45], it is a typical water bearing clay mineral and it is associated with the bending vibrations of structural water molecules. For the averaged spectrum for all the samples associated with Halloysite shown in Fig. 4 (subplot c) has hydroxyl stretching vibrations at 3698, 3672, 3655 and 3622 cm^− 1^ in the 3800–3000 cm^− 1^ regions. The characteristic bands between 1750 and 600 cm^− 1^, which includes smaller sharp peaks at 1020 and 920 cm^− 1^ can be said to be due to the alumino-silicate lattice vibrations and Al-OH deformation vibrations [45] respectively like in the case of kaolinite minerals which exhibit similar spectral characteristics to Halloysites. Two more bands observed at 1650 and 1530 cm^− 1^ can be assigned to water bending modes and C–H in-plane bending vibration.

**Fig. 5:**
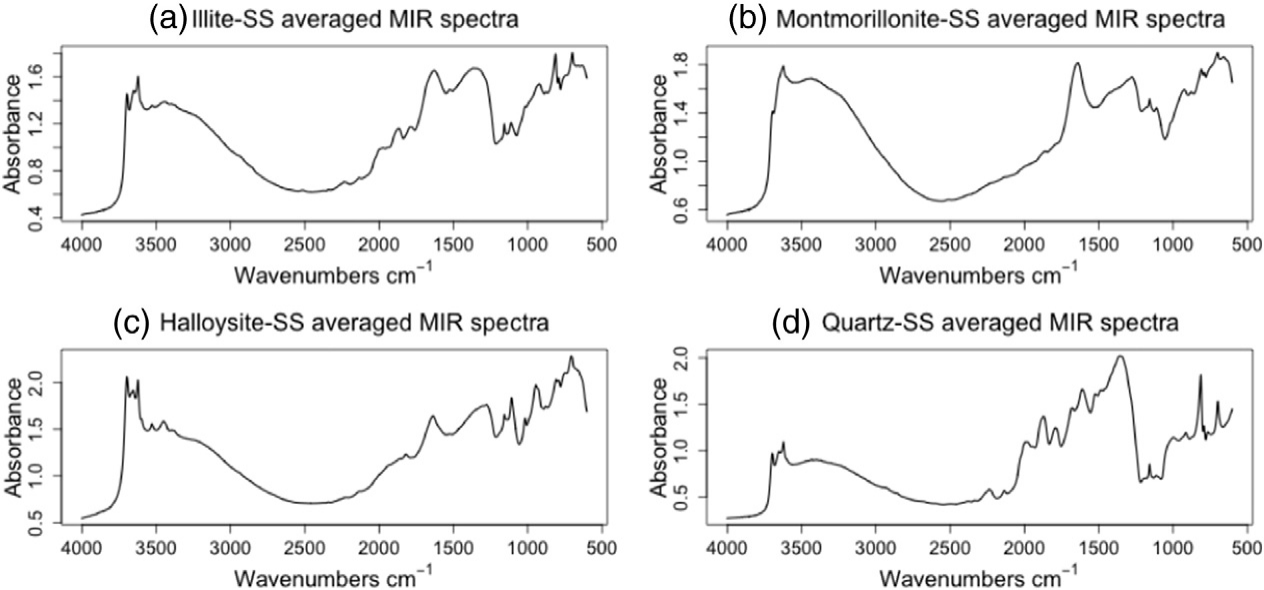
HQISS averaged MIR spectra per subspace.

Finally, the averaged spectrum representing the soils found to be spectrally close to quartz pure mineral spectrum as shown in Fig. 5 (subplot d) shows intense peaks in the regions 2000–1650 and 1080–700 cm^− 1^ [24]. The fundamental O–Si–O stretching and bending frequencies at 1080, 800–780 and 700 cm^− 1^ were found to be the most dominant bands in the infrared spectra of quartz-rich soils. In our study, we observed other two prominent peaks outside these regions at 1350 and 1220 cm^− 1^, which are dominated by C–H bending vibrations from organic materials.

**Fig. 6:**
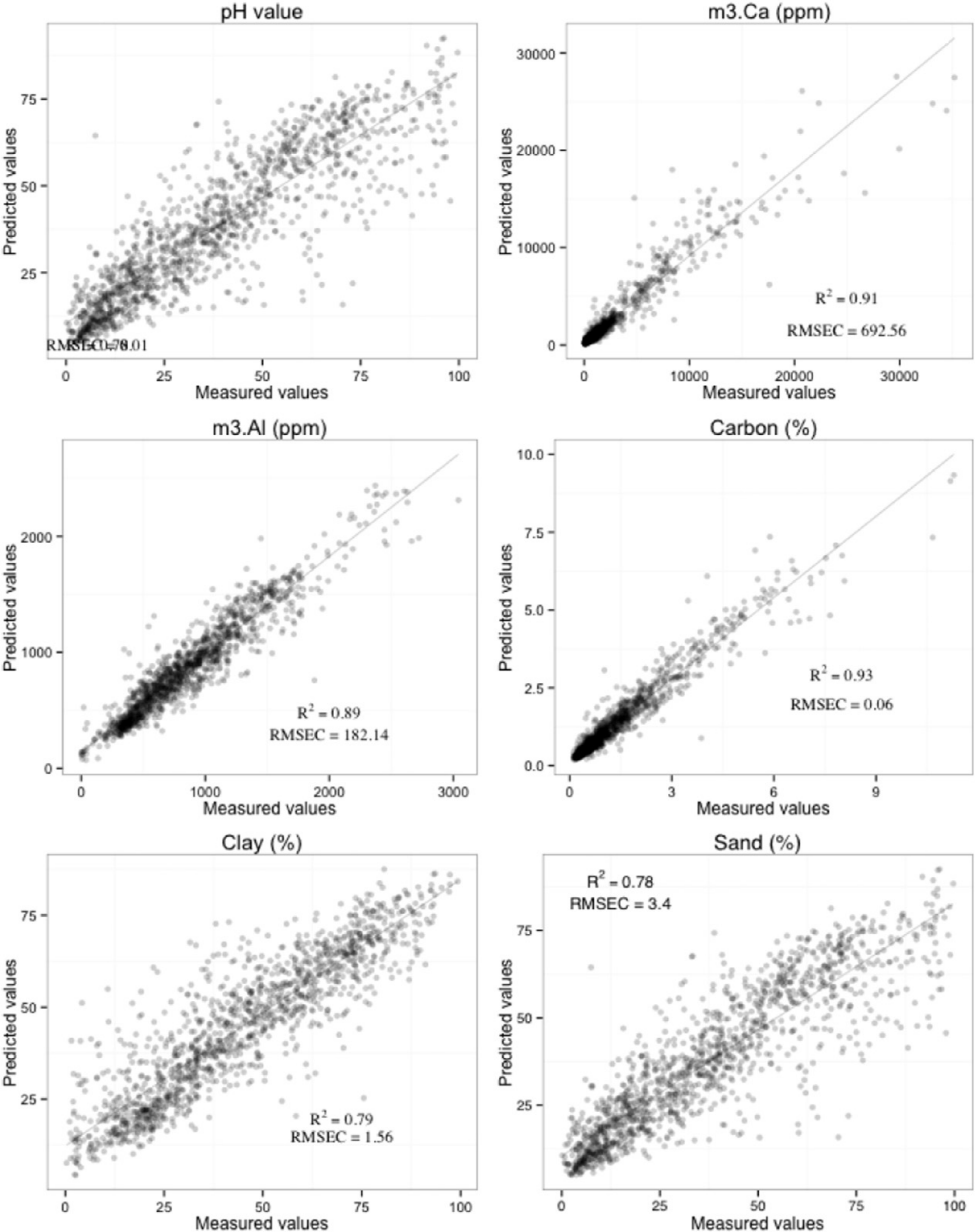
Figure linear regression for the calibration set (n = 1325) of predicted against measured soil property values (r^2^, a coefficient of determination; RMSEC, root mean square error of calibration) using 1st derivative spectra.

**Fig. 7:**
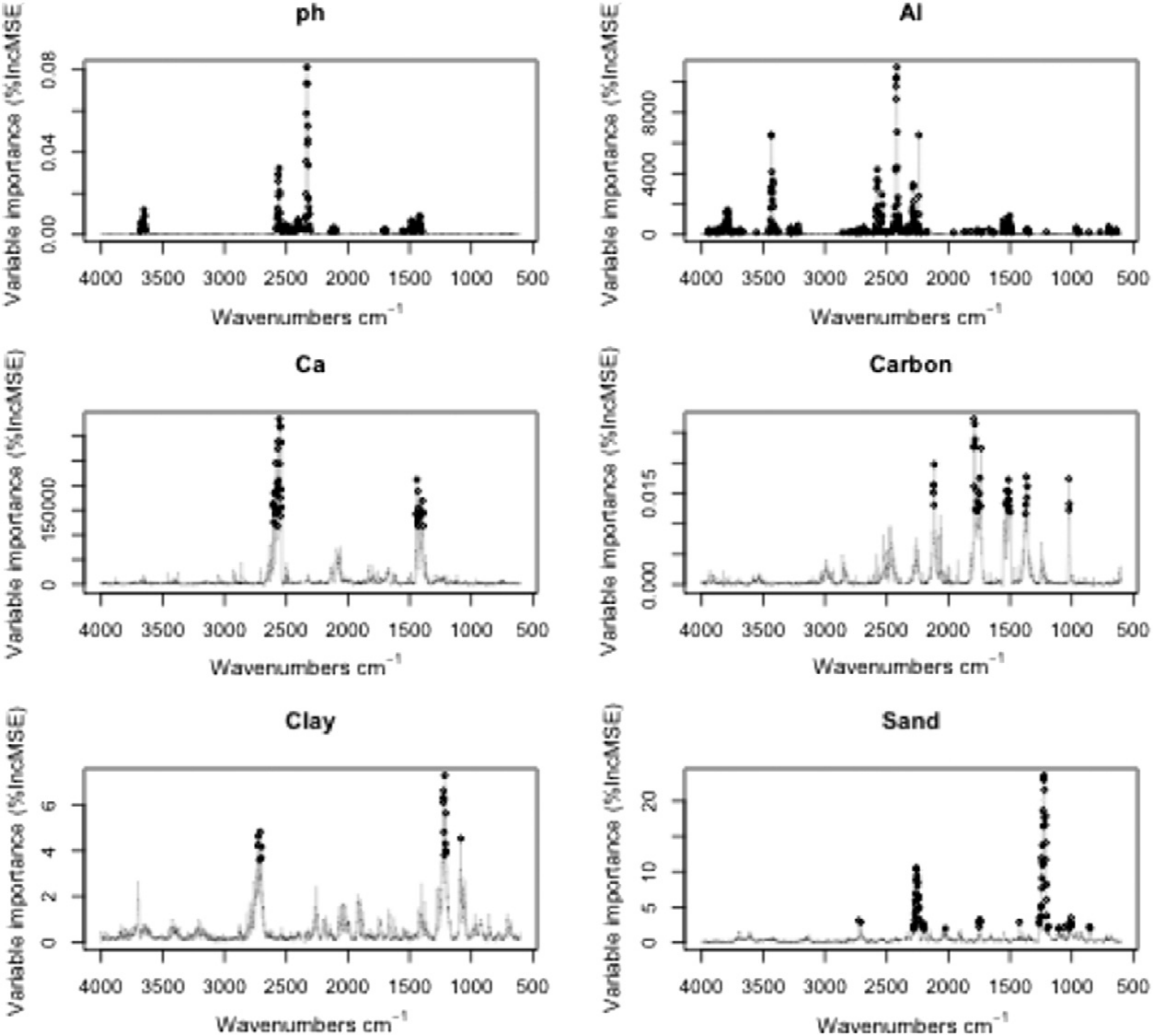
1st derivative MIR spectra important wavebands for predicting pH, m3.Ca. m3.Al, Carbon, Clay and Sand. The shaded points highlight all the important variables tried at each split for each model (pH = 182; m3.Al = 388; m3.Ca = 40; Total Carbon = 40; Clay = 19 and Sand = 86).

### 3.3 Random forests ensemble tree regression models

Fig. 6 gives scatter plots for the global calibration models showing predicted values against the actual measurement values. Similar scatter plots were found for archetype subspaces but with lower r^2^ and higher RMSE values. We have not shown the scatter plot for the combined archetype models. Our results showed that the best RF model combinations for the Savitzky–Golay 1st derivative processed spectra are to be 500 trees but different numbers of random variables were tried at each split in the six calibration models (pH = 182; m3.Al = 388; m3.Ca = 40; total carbon = 40; clay = 19 and sand = 86). A similar number of trees was reported by McDowell et al. [22] for soil total carbon analysis using MIR data for 307 Hawaiian soil samples. But, their model used up to 396 random variables, which are about 10 times the number of variables, used in this study for total carbon.

Soil pH was well calibrated (r^2^ = 0.87 and RMSEC = 0.01). The result was as good as obtained by Terhoeven-Urselmans et al. [37] for the prediction of soil properties from a globally distributed soil MIR spectral library of 971 soil samples (r^2^ = 0.81, RMSEC = 0.63). Similar results were also reported by Shepherd and Walsh [32] for the characterization of soil properties from a spectral library with 758 soils from eastern and southern Africa (r^2^ = 0.83, RMSEC = 0.34). But, in terms of RMSEC, our results are much better from those previously reported. However, our model seems to overestimate alkaline soil samples, which can be attributed to fewer samples in this range. There were 182 wavebands found to be the most significant in predicting soil pH. These wavebands are 3683–3639; 2580–2306–; 2137–2098; 1709–1689; 1556–1400 cm^− 1^ (Fig. 7). These bands are associated with hydroxyl stretching vibrations, alumino-silicate lattice vibrations and Al-OH deformation vibrations [45] and very similar to the ones found by Terhoeven-Urselmans et al. [37] using a PLSR model.

Both m3.Al and m3.Ca were satisfactorily calibrated with the MIR spectra (r^2^ = 0.89 and RMSEC = 182.14; r^2^ = 0.91 and RMSEC = 692.56;) respectively. The relatively high cross-validated RMSEC for m3.Ca can be attributed to the few points with high m3.Ca values which were under-predicted by MIR. A total of 388 important variables were reported for a m3.Al which occurred almost across the full MIR spectra range, from 3950 to 3664; 3554–3209; 2858–2173; 1957, 1871–1344; 1205; 962–632. These bands were mainly concentrated in the parts of the spectrum associated with Si–O–H vibration of clays, kaolinite and Fe oxides at 3719–3685 cm^-1^, O-H stretching of Gibbsite at the bands 3525–3460 cm^− 1^ and a small peak at 920 cm^-1^ associated with Al-OH deformation of kaolinite [24], [38].

Soil total carbon was predicted well for the calibrations set (r^2^ = 0.93; RMSEC = 0.06). Terhoeven-Urselmans et al. [37] reported a lower accuracy (r^2^ = 0.77) for similar diverse calibrations samples while McDowell et al. [22] reported higher accuracy (r^2^ = 0.96) but with a large RMSEC probably due to a wide range of total carbon in the calibration set (0.24 to 55.29%) compared to (0.11 to 11.3%) of total carbon used in this study. Important wavebands for total carbon were 40, from 2121 to 2114; 1794–1736; 1537–1500; 1375–1360; 1022–1018 cm^− 1^ Fig. 7. These are the ranges associated with C=O stretching [38] at 1775–1711 cm^− 1^ and 1350–1550 regions which contain absorption mainly resulted from soil calcium carbonate, and a stronger absorption meant a higher calcium carbonate content and a higher soil pH [12].

Predictions for particle size were good, for clay (r^2^ = 0.79; RMSEC = 1.56) and for sand (r^2^ = 0.78; RMSEC = 3.4). However, the sand random forest regression tended to under-predict sand content for samples with actual measurement of sand > 50% samples while samples with clay < 50% were over predicted Fig. 6. Our results were broadly similar to those of previous researchers [26], [37] in terms of r^2^ values but with higher RMSEC values than those obtained in this study. Important wavebands for clay were 2731–2700; 1228–1205; 1084 cm^− 1^, which mainly correspond to quartz and other clay minerals [18] which also overlapped with important variables found in sand prediction. Additional wavebands in the regions 2285–2025, 1751–174 and 1423 cm^− 1^ were found to be important for sand prediction, which corresponds to alumino-silicate lattice vibrations and Al-OH deformation vibrations [45].

### 3.4 Model predictive performance

Validation statistics calculated from both global and subspace models show that 1st and 2nd derivative processed spectra gave the best models with highest Q^2^ values. Q^2^ is the cross-validated r^2^ [44] for the independent validation set. Mehlich-3 Ca model gave poor predictions Q^2^ < 0.6 except for the HQISS 1st derivative processed spectra and CACSS 2nd derivative processed spectra subspace models, Table 5. Few points with high m3.Ca could have caused the poor calcium models. Although, MSC preprocessed spectra calibrated well with most of the soil properties they gave low Q^2^ indicating that the predictions were so poor, and do not predict better than chance. Clay and sand models gave stable predictions for the 1st derivative preprocessed spectra. Combining SNV and detrending did not give better predictions than SNV only.

**Table 5:** Calibration and validation (independent samples) sets' R^2^ and Q^2^ values. C refers to total soil carbon. No archetypes generated for 2nd derivatives spectra.

Subspace	Preprocessing	R^2^ for calibration set; n = 1329						Q^2^ for validation set; n = 575					
		pH	m3.Al	m3.Ca	C	Clay	Sand	pH	m3.Al	m3.Ca	C	Clay	Sand
HQI	First derivative	0.86	0.88	0.91	0.93	0.78	0.77	0.60	0.82	0.59	0.86	0.80	0.83
	Second derivative	0.90	0.90	0.91	0.92	0.80	0.79	0.73	0.79	0.65	0.85	0.78	0.84
	Msc	0.81	0.82	0.89	0.90	0.74	0.70	0.10	0.50	0.10	0.10	0.63	0.60
	SNV	0.80	0.96	0.98	0.98	0.93	0.83	0.33	0.69	0.10	0.75	0.81	0.82
	SNV_Detrend	0.80	0.82	0.89	0.92	0.74	0.72	0.35	0.73	0.10	0.77	0.78	0.81
Cosine	First derivative	0.83	0.85	0.90	0.88	0.74	0.73	0.61	0.84	0.50	0.85	0.75	0.78
	Second derivative	0.85	0.86	0.89	0.89	0.78	0.77	0.69	0.83	0.62	0.79	0.75	0.78
	Msc	0.79	0.81	0.89	0.89	0.73	0.72	0.10	0.10	0.10	0.10	0.43	0.34
	SNV	0.78	0.80	0.88	0.87	0.73	0.72	0.55	0.76	0.10	0.63	0.73	0.73
	SNV_Detrend	0.79	0.82	0.88	0.91	0.74	0.72	0.44	0.72	0.22	0.78	0.73	0.74
SOM	First derivative	0.85	0.88	0.91	0.92	0.79	0.78	0.59	0.78	0.41	0.86	0.79	0.80
	Second derivative	0.89	0.90	0.91	0.92	0.80	0.80	0.71	0.81	0.54	0.84	0.79	0.82
	Msc	0.80	0.81	0.90	0.91	0.74	0.72	0.10	0.06	0.10	0.40	0.46	0.61
	SNV	0.81	0.81	0.89	0.92	0.74	0.71	0.53	0.77	0.55	0.81	0.76	0.76
	SNV_Detrend	0.80	0.81	0.89	0.93	0.74	0.71	0.46	0.77	0.57	0.80	0.75	0.77
Archetype	First derivative	0.87	0.89	0.91	0.93	0.79	0.78	0.60	0.79	0.41	0.87	0.79	0.80
	Second derivative	–	–	–	–	–	–	–	–	–	–	–	–
	Msc	0.80	0.82	0.89	0.91	0.75	0.72	0.10	0.06	0.10	0.40	0.47	0.61
	SNV	0.81	0.83	0.89	0.90	0.74	0.71	0.53	0.79	0.55	0.79	0.76	0.76
	SNV_Detrend	0.80	0.82	0.88	0.93	0.76	0.73	0.46	0.78	0.56	0.80	0.77	0.79
All	First derivative	0.87	0.89	0.91	0.93	0.79	0.78	0.62	0.84	0.29	0.78	0.79	0.81
	Second derivative	0.74	0.85	0.67	0.86	0.80	0.81	0.52	0.80	0.21	0.72	0.80	0.84
	Msc	0.82	0.83	0.90	0.92	0.75	0.72	0.10	0.17	0.10	0.26	0.45	0.39
	SNV	0.82	0.83	0.89	0.92	0.75	0.72	0.58	0.78	0.28	0.77	0.75	0.75
	SNV_Detrend	0.81	0.84	0.89	0.93	0.75	0.73	0.49	0.78	0.29	0.80	0.76	0.76

Additional results showed RMSE values obtained from the independent validation set using subspace and global models are given in Table 6. In general, we found predictions from the global models outperformed subspace models in many instances except in a few of them. Sand and clay RMSE values from ArchetypeSS are > 50% higher than all the sand and clay global models except for the MSC preprocessed spectra which were lower < 12%.

**Table 6:** Independent holdout validation set RMSE values for local and global RF models^1^.

Global/Local	Preprocessing method	pH	m3.Al	m3.Ca	Total carbon	Clay	Sand
CACSS	First derivative	0.6	177.77	998.58	0.43	12.5	12.94
	MSC	**1.93**	485.64	6042.29	1.23	**18.48**	22.36
	Second derivative	0.53	180.77	868.19	0.51	12.72	13.07
	SNV	0.65	215.27	1561.67	0.67	13.11	14.46
	SNV + Detrend	0.72	232.86	1242.18	0.52	13.02	14.08
HQISS	First derivative	0.61	189.67	902.61	0.42	11.39	**11.36**
	MSC	**1.61**	**366.5**	4943.63	1.24	**18.16**	**20.27**
	Second derivative	0.5	182.29	**726.07**	0.41	11.71	11.2
	SNV	0.66	216.99	1177.52	0.52	12.76	13.96
	SNV + Detrend	**0.67**	217.83	1243.51	0.51	12.61	13.98
Archetype-SS	First derivative	0.52	321.11	1116.33	0.42	18.25	22.77
	MSC	**0.59**	**389.06**	**1269.76**	0.64	20.35	24.18
	SNV	0.56	360.29	1533.73	0.73	20.15	24.12
	SNV + Detrend	**0.6**	370.35	1478.75	0.65	20.37	24.04
SOM-SS	First derivative	0.62	209.12	1078.02	**0.41**	11.46	12.33
	MSC	**1.25**	428.17	**3311.35**	0.86	**18.6**	**17.36**
	Second derivative	0.52	192.98	954.05	0.45	11.48	**11.67**
	SNV	0.66	212.3	941.39	0.48	**12.29**	13.69
	SNV + Detrend	0.71	212.27	**923**	0.5	12.67	**13.37**
*Global*	*First derivative*	*0.60*	*164.42*	*888.38*	*0.42*	*11.42*	*11.51*
	*MSC*	*2.09*	*403.42*	*5711.57*	*0.95*	*18.71*	*21.68*
	*Second derivative*	*0.49*	*169.8*	*813.06*	*0.41*	*11.27*	*12.18*
	*SNV*	*0.63*	*205.32*	*1190.2*	*0.53*	*12.56*	*13.88*
	*SNV* + *Detrend*	*0.69*	*208.9*	*1188.51*	*0.49*	*12.28*	*13.57*

1 Bold figures show local models, which are better than global models. Global model values have been italicized for clarity.

SOMSS models predicted sand content much better with lower RMSE values than the global one except for the 1st derivative preprocessed spectra. The second best-predicted soil property using the SOMSS is m3.Ca, which had lower RMSE value except for the 1st and 2nd derivative preprocessed spectra. Local models for m3.Al and total carbon mostly gave high RMSE values compared to the global models. Although the CACSS local models were poor compared to the global ones with RMSE values in the range of 2–30%, pH model for the 1st derivative preprocessed spectra gave RMSE value equal to the RMSE for the global model. However, MSC processed spectra in the CACSS gave lower RMSE value of about 8% lower than the global one. 1st derivative preprocessed spectra gave total carbon local models with RMSE values in the range, 0.41–0.43 which is almost equal to the global model with RMSE of 0.42. RMSE values from the MSC preprocessed spectra were the highest among the five spectral preprocessing methods in both the global and local models. This seems to agree well with previous work done for modeling soil carbon fractions using visible near-infrared and mid-infrared spectroscopy [19] but contradicts previous work [8] who used partial least squares (PLS) regression to predict soil organic carbon using near-infrared spectra. In summary, total carbon, clay and sand gave stable modes while pH, m3.Al and m3.Ca gave models with poor predictive performance. Based on these results it is possible that the type of analytical method for acquiring soil properties measurements data influences model predictive performance. Because it is beyond the scope of this current study we suggest that methods for minimizing or controlling analytical measurement errors should be investigated.

## 4 Conclusion

We did not find evidence in these results to support the main hypothesis of this study. We, therefore, conclude that global models are more accurate than the local ones. Although our findings are at variance with other reported work [42]. However, Ramirez-Lopez et al. and Sankey et al. [28], [30] got similar results to ours and concluded that global models predicted the validation dataset better than the local ones. Spectral data processing using Savitzky and Golay algorithm outperformed the other methods with the 2nd derivative giving the best models for pH, m3.Ca, total carbon and clay while the 1st derivative method gave the best models for m3.Al and sand. On the other hand, MSC preprocessed spectra gave predictions with largest RMSEP values relative to all the other methods. This means that MSC preprocessed spectra may have a larger signal to noise ratio either caused by the removal of valuable information or the method was unable to filter out all the irrelevant information. We therefore suggest that future studies need not to use MSC as the only spectral preprocessing method because it may lead to models with low predictive accuracy. The ability of the HQISS to group soil MIR spectra according to how they are similar to the four pure mineral spectra confirms MIR spectral signatures are due to vibrations of molecular groups within minerals and organic molecular groups [18]. Since the CACSS did not form well-separated clusters within the local models we suggest future research to consider modifying the method and include only the most informative regions known to contain mineral figure print. Also we recommend further testing of our proposed method to search for local subspaces in large spectral libraries. Other different model fitting methods like support vector machine neural networks and boosted regression trees may be worthy to be tested in a similar setup like for this study.

## Conflict of interest

None.
